# Linking community assembly and structure across scales in a wild mouse parasite community

**DOI:** 10.1002/ece3.5785

**Published:** 2019-12-09

**Authors:** Evelyn C. Rynkiewicz, Andy Fenton, Amy B. Pedersen

**Affiliations:** ^1^ Department of Science and Mathematics Fashion Institute of Technology State University of New York New York NY USA; ^2^ Institute of Evolutionary Biology & Centre for Immunity, Infection and Evolution School of Biological Science University of Edinburgh Edinburgh UK; ^3^ Institute of Integrative Biology University of Liverpool Liverpool UK

**Keywords:** *Bartonella*, coinfection, community assembly, community structure, *Eimeria*, helminths, multi‐state Markov model, nestedness, wild mice

## Abstract

Understanding what processes drive community structure is fundamental to ecology. Many wild animals are simultaneously infected by multiple parasite species, so host–parasite communities can be valuable tools for investigating connections between community structures at multiple scales, as each host can be considered a replicate parasite community. Like free‐living communities, within‐host–parasite communities are hierarchical; ecological interactions between hosts and parasites can occur at multiple scales (e.g., host community, host population, parasite community within the host), therefore, both extrinsic and intrinsic processes can determine parasite community structure. We combine analyses of community structure and assembly at both the host population and individual scales using extensive datasets on wild wood mice (*Apodemus sylvaticus*) and their parasite community. An analysis of parasite community nestedness at the host population scale provided predictions about the order of infection at the individual scale, which were then tested using parasite community assembly data from individual hosts from the same populations. Nestedness analyses revealed parasite communities were significantly more structured than random. However, observed nestedness did not differ from null models in which parasite species abundance was kept constant. We did not find consistency between observed community structure at the host population scale and within‐host order of infection. Multi‐state Markov models of parasite community assembly showed that a host's likelihood of infection with one parasite did not consistently follow previous infection by a different parasite species, suggesting there is not a deterministic order of infection among the species we investigated in wild wood mice. Our results demonstrate that patterns at one scale (i.e., host population) do not reliably predict processes at another scale (i.e., individual host), and that neutral or stochastic processes may be driving the patterns of nestedness observed in these communities. We suggest that experimental approaches that manipulate parasite communities are needed to better link processes at multiple ecological scales.

## Graphical abstract

Many wild animals are simultaneously infected by multiple parasite species, and so host–parasite communities can be a valuable tool to investigate connections between community structure at multiple scales, as each host can be considered to contain a replicate parasite community. Here, we combine analyses of community structure and assembly at both the host population and individual scales using an extensive dataset on wild wood mice (*Apodemus sylvaticus*) and their parasite community. While our results show that these parasite communities are not randomly assembled, patterns at one scale (i.e., host population) do not reliably predict processes at another scale (i.e., individual host).

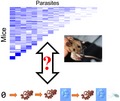

## 1 INTRODUCTION

Ecological systems are fundamentally hierarchical, from individuals, to populations, communities, and the broader ecosystem. A major challenge in ecology is to understand the extent to which processes at one scale (e.g., within a population) affect patterns and processes at another (e.g., across the community). Specifically, a key issue is to investigate how ecological communities assemble and the extent to which observed community composition reflects underlying processes occurring at finer scales. To assess the connection between community assembly and structure, we need empirical systems at which processes at distinct scales can be quantified, and for which a large number of replicates can be sampled. Within‐host–parasite communities have recently been suggested to have potential for developing our understanding of the processes underlying community assembly and structure (Blackwell, Martin, Kaplan, & Gurven, 2013; Cobey & Lipsitch, 2013; Costello, Stagaman, Dethlefsen, Bohannan, & Relman, 2012; Dallas & Cornelius, 2015; Dallas, Park, & Drake, 2016). While host–parasite systems carry some important differences to free‐living systems, such as habitat patches being mobile (in the case of animal hosts) and the host being an evolving habitat and food resource (Johnson, De Roode, & Fenton, 2015; Poulin & Valtonen, 2001; Seabloom et al., 2015; Ulrich, Almeida, & Gotelli, 2009), the typically large number of communities (infected hosts) and relative ease of longitudinal study of successive infections within individual hosts provides a great opportunity to study the assembly of multiple replicate communities in an easily observable timespan.

Parasites are extremely common in nature, and most wild hosts are coinfected by multiple parasite species (defined here to include both macroparasites (e.g., helminths and ectoparasites) and microparasites (e.g. viruses, bacteria, protozoans)) simultaneously and/or sequentially throughout their life (Poulin, 1996). Each individual host can therefore be considered an ecosystem, with many habitats for parasites and pathogens to infect, forming a clearly defined within‐host ecological community (Pedersen & Fenton, 2007; Restif & Graham, 2015; Rynkiewicz, Pedersen, & Fenton, 2015). Furthermore, host–parasite systems are inherently hierarchical; each host is infected with its own community of parasites, and these hosts are linked by potential dispersal via parasite transmission (Mihaljevic, 2012). Hence, both extrinsic (between‐host) factors, such as parasite exposure or variation in parasite species abundance, and intrinsic (within‐host) factors, such as host immune function and interactions between coinfecting parasite species, can combine to influence community structure at multiple scales (Joseph, Mihaljevic, Orlofske, & Paull, 2013; Lima, Giacomini, Takemoto, Agostinho, & Bini, 2012; Poulin, 2001; Ulrich & Gotelli, 2007; Zelmer & Arai, 2004). Therefore, processes occurring at one scale can impact patterns and processes at another scale. For example, treating to reduce the burden of gastrointestinal worms in individual African buffalo increased the survival of treated hosts, which could exacerbate the invasion and spread of bovine TB at the host population scale (Ezenwa & Jolles, 2015). Scaling down, individual host and vector risk for infection with the agent of Lyme disease is influenced by the diversity and composition of the wider host community (Ostfeld & Keesing, 2000). It remains an open question to what extent patterns of community structure (e.g., community composition) at one scale reflect processes (e.g., assembly order) at another. The hierarchical nature of host–parasite systems, enabling measurements of between‐host community composition to be coupled with data on community assembly (infection order) within individual hosts, may provide a means to address this question.

To investigate the relationship between the structure of within‐host–parasite communities and their assembly patterns over time, we used wild wood mice, *Apodemus sylvaticus*, and their species‐rich endoparasite community. These datasets comprised longitudinal data (capture–mark–recapture) on individually tagged mice, where infection with over 30 taxonomically diverse parasite species was measured through time. These extensive within‐host–parasite community data allow for the quantification of the assembly order of within‐host–parasite communities for each individual over the course of their life and across the same population over time. To analyse parasite community structure at the host population scale, we used a nestedness analysis approach. Nestedness describes the structure and co‐occurrence of species in a community, testing if less rich communities are perfect subsets of richer ones (Atmar & Patterson, 1993). Nested communities can arise when some species rely on others for survival or reproduction, such as mutualisms, food web, or trophic interactions, from neutral processes‐like variation in species abundance, patch colonization history, or through stochastic colonization or extinction, which can be influenced by variation in species abundance or patch quality (Amundsen et al., 2009; Bracken, Friberg, Gonzalez‐Dorantes, & Williams, 2008; Calatayud, Madrigal‐Gonzalez, Gianoli, Hortal, & Herrero, 2017; McQuaid & Britton, 2013; Ulrich et al., 2009). Notably, it has been suggested that nestedness in a community can imply a fixed order of colonization or extinction which structures communities in a predictable way (Diamond, 1975; Ulrich et al., 2009), and nestedness theory has been used to analyze predictable species loss or gain from islands or isolated patches (Atmar & Patterson, 1993; Ulrich et al., 2009).

In the context of host–parasite systems, nestedness analyses have previously been used to demonstrate significant structure of parasite communities in fish (Lima et al., 2012; Poulin & Valtonen, 2002) and amphibian populations (Johnson & Hoverman, 2012). These findings support epidemiological theory (e.g., Dobson, 1990) which predicts that parasite communities may tend to show nested structures, with certain “core” species (typically those with high basic reproduction ratios, *R*_0_) tending to be found in all communities, whereas “satellite” species (those with lower *R*_0_ values) will typically be much rarer (Bush & Holmes, 1986; Holmes & Price, 1986; Stock & Holmes, 1987). Furthermore, it is well known that there is likely to be a strong link between a parasite's *R*_0_, its population‐level prevalence, and the average age at which hosts first become infected with that parasite (Anderson & May, 1991). This is similar to the pattern predicted by variation in the dispersal ability of species in their ability to move to new habitat patches in free‐living systems (Leibold et al., 2004). Bringing these ideas together, we hypothesized that the patterns of community nestedness observed at the host population level should be predictive of the order of parasite assembly (i.e., infection order) at the individual host level (Götzenberger et al., 2012; Lima et al., 2012; Lindo, Winchester, & Didham, 2008). We tested this hypothesis using cross‐sectional (population scale nestedness) and longitudinal data (individual scale order of infection) in the same populations of wild mice and their parasites. If the order of community assembly at the individual scale matches the predictions based on community structure at the population scale, then we can conclude that patterns of nestedness at one scale predict the process of community assembly order at the other.

## 2 METHODS

### 2.1 Sample collection

All parasite samples were collected from wild wood mice at three sites near Liverpool, UK: Haddon Wood (N 53 2716°, E −3 0297), Manor Wood (N 53 3301°, E −3 0516°), and Rode Hall (N 53 1213°, E −2 2798°). There were five 70 × 70m grids among the sites, where each grid had 64 trap stations (10 m apart), with 2 Sherman live traps (2 × 2.5 × 6.5‐inch folding trap, H.B. Sherman) at each trapping station, 128 traps per grid (Knowles, Fenton, & Pedersen, 2012; Withenshaw, Devevey, Pedersen, & Fenton, 2016). The traps were baited at dusk with crimped oats and carrot; bedding was also placed in the trap as nesting material. The following morning, all mice were given a numbered subcutaneous microchip transponder (PIT tag) at first capture. Fecal and small volume blood samples were collected from each individual once per trapping session. Gastrointestinal (gut) parasite infections (helminth worms and coccidial protozoans) were identified to species, and burdens were measured (either fecal egg or oocyst counts (FEC/FOC), respectively) using salt flotation and microscopy. Infection with blood parasites (e.g., *Bartonella* spp., a flea‐transmitted bacterium, and *Trypanosoma grosi*, a flea‐transmitted protozoan) was identified using targeted, nested PCR assays on DNA extracted from blood (for more details on these methods see Knowles et al., 2013; Withenshaw et al., 2016). Our previous research on these wild rodent and parasite communities has shown that most parasites are host‐specific (Knowles et al., 2012; Withenshaw et al., 2016), therefore, we focused our analysis on the parasite communities in wood mice only.

Trapping took place between May and December across 4 years (2009–2012). In 2009–2011, the grids were sampled every 4 weeks, while in 2012, grids were sampled every 2 weeks. This resulted in a per year effort of 5,760 trap nights per year in 2009–2011 and 11,520 trap nights in 2012. Data from 2009 to 2011 and 2012 were considered separately due to these differences in sampling regimes. The 2012 dataset had more repeat captures of individuals and thus made it more suitable for longitudinal, individual‐scale analyses, while the 2009–2011 datasets are better suited for cross‐sectional, population‐scale analyses. The unique nature of these datasets, with extensive longitudinal and cross‐sectional data on the same populations of wild wood mice, gives us the ability to directly compare predictions of parasite community assembly based on population prevalence and order of infection likelihood to determine the concordance of population‐ and individual‐scale patterns of parasite community structure.

### 2.2 Parasite community data

To test for nestedness at the population scale, we used data from the first capture of each individual to avoid pseudoreplication due to repeat captures of the same mouse. In addition, we only included parasite species that were commonly found in all 4 years of sampling, which resulted in 16 parasite species for 2009–2011 and 15 for 2012 (Table 1). For tests of community assembly at the individual scale, we analyzed longitudinal data limited to records with multiple captures per individual (2+ captures) to assess the temporal order of parasite species infection throughout each individual host's life. These analyses used either all parasites from the population‐level analyses for a coarse assessment of individual‐scale host–parasite community assembly (rank order analysis; see below) or the three most abundant species for a finer‐resolution assessment (multi‐state Markov models; see below).

**Table 1:** Total number and infection prevalence for each parasite species in the wild wood mouse populations in the 2009–2011 and 2012 datasets

Parasite	Taxon	Infection site	Prevalence (number infected)	
			2009–2011	2012
*Aspiculuris* sp.	Helminth	Gut	0.1 (13)	0.028 (9)
*Bartonella birtlesii*	Bacteria	Gut	0.094 (127)	0.236 (76)
*Bartonella doshiae*‐like	Bacteria	Blood	0.042 (57)	0.087 (28)
*Bartonella grahamii*	Bacteria	Blood	0.196 (266)	0.102 (33)
*Bartonella taylorii*	Bacteria	Blood	0.233 (316)	0.317 (102)
*Bartonella* type BGA	Bacteria	Blood	0.01 (13)	0.078 (25)
*Capillaria* sp.	Helminth	Gut	0.024 (33)	0.016 (5)
*Cestodes* spp.	Helminth	Gut	0.051 (69)	0.292 (94)
*Eimeria apionodes*	Protozoan	Gut	0.217 (295)	0.134 (43)
*Eimeria hungaryensis*	Protozoan	Gut	0.284 (386)	0.18 (58)
*Eimeria* sp. 1	Protozoan	Gut	0.11 (150)	0.016 (5)
*Eimeria* sp. 2	Protozoan	Gut	0.008 (11)	0.019 (6)
*Eimeria uptoni*	Protozoan	Gut	0.007 (9)	NA
*Heligmosomoides polygyrus*	Helminth	Gut	0.33 (452)	0.224 (72)
*Syphacia stroma*	Helminth	Gut	0.06 (81)	0.56 (18)
*Trypanosoma grosi*	Protozoan	Blood	0.102 (138)	0.068 (22)

### 2.3 Population scale—nestedness of parasite communities

To analyse the differences between observed and null model communities, the within‐host–parasite communities were arranged in an incidence matrix of individual hosts (“sites”) and parasite species (“species occupying those sites”) and analyzed using nestedness of overlap and decreasing fill (NODF) method; this was implemented by the “oecosimu” function in the “vegan” package (Oksanen et al., 2013) in R (R Core Team, 2018). The nestedness matrix is the most efficient “packing” of hosts and parasites, with the most abundant parasite species in the left column and the most highly parasitized host (highest parasite species richness) in the top row, with the others hosts and parasites “ordered in a manner to minimize unexpected species absences and presences” (Atmar & Patterson, 1993, p 375).

Three null models were constructed to correspond to alternative hypotheses of intrinsic (host individual level)‐ or extrinsic (parasite identity or characteristics)‐based mechanisms underlying the community assembly process (Almeida‐Neto, Guimaraes, Guimaraes, Loyola, & Ulrich, 2008): (a) completely random, where parasites were randomly assigned to hosts irrespective of host or parasite identity (i.e., the same number of parasites is present in the null model as in the observed community, but individual host (patch) species richness and parasite abundance are drawn at random from the entire community), (b) random with respect to host identity, which tests for whether population‐level patterns are driven by individual (intrinsic) mechanisms influencing host (patch) quality or exposure (i.e., parasite species richness in each host (row totals) is the same as in the observed community, but the species in each community are drawn at random), and (c) random with respect to parasite species identity to test whether extrinsic mechanisms drive parasite co‐occurrence patterns, such as parasite species identity or abundance (i.e., parasite abundance (column totals) in the null model is the same as in the observed community but parasites are assigned to hosts at random) (see Figure S1 for a visual example of each null model). Testing these null models provides information about likely mechanisms structuring the overall parasite community, that is, host individual‐level (intrinsic) variation, population‐level (extrinsic) variation in parasite abundance, both, or neither. One hundred simulated null communities were constructed for each method to test against each dataset of observed parasite infection in wild wood mice. The datasets used included the following: (a) the combined three‐year dataset (2009–2011), (b) each year of that dataset individually, and (c) the dataset from 2012, in order to compare population‐ and individual‐level community assembly between years. We also analyzed nestedness in adult hosts and young hosts (juveniles and sub‐adults) to test whether parasite communities became more nested as hosts aged. Data from all trapping grids were pooled in order to have as large a sample size as possible for testing against the null models, while we recognize that there is possibly variation in parasite exposure between grid locations.

### 2.4 Individual host scale—order of parasite infection

The nestedness analyses suggested that parasite community structure at the host population scale was primarily driven by aspects relating to parasite species identity, such that there were highly prevalent “core” species found in most communities, and less prevalent “satellite” species occurring in fewer communities (see Results). As described previously, epidemiological theory suggests there should be a strong link between a parasite's prevalence and the average age at which hosts first become infected with that parasite (Anderson & May, 1991; see also Supporting Information for a simulation model of this relationship; Figure S2). We therefore hypothesized that nestedness “rank” of each parasite in the nested matrix would be predictive of the order of parasite assembly (i.e., infection order) at the individual host scale. We tested these predictions with two analyses at the individual host scale using longitudinal parasite community assembly data.

**Figure 1:**
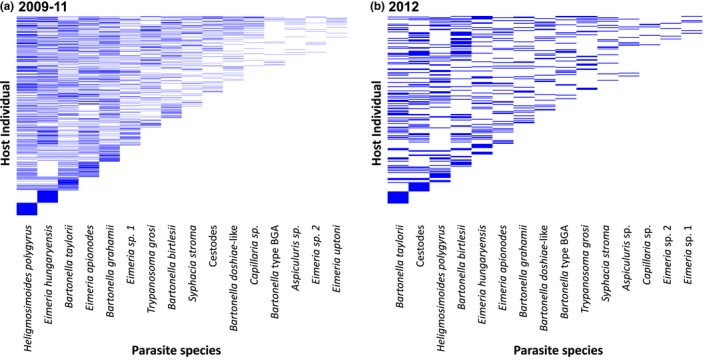
Nestedness matrices for the parasite community used in the analysis of the (a) 2009–2011 dataset and (b) the 2012 dataset. Each row in the *y*‐axis represents an individual host with all parasites included in the analyses along the *x*‐axis. A horizontal line represents if the host is infected with a parasite. In each nestedness matrix, the host coinfected with the most parasites is located in the top row and the most abundant parasite is located in the left column. The rest of hosts and parasites are then arranged to minimize unexpected species presences or absences (i.e., to create the most efficiently packed matrix). All data used in nestedness analyses were from a host's first capture

First, we carried out a nonparametric analysis of ranks (Spearman's rank) on all parasite species, to analyse the concordance between the predicted rank order of infection from the nestedness analysis at the host population scale against the observed rank order of infection for each individual host. For example, using the nestedness matrix from the 2009 to 2011 combined dataset (Figure 1a), the parasite predicted to infect first is *Heligmosimoides polygyrus* (rank = 1), predicted second to infect is *Eimeria hungaryensis* (rank = 2), predicted third to infect is *Bartonella taylorii* (rank = 3), etc. To calculate observed rank orders of infection, longitudinal data were organized by host individual and date of capture to rank when each parasite infected the host over the course of the host's lifetime. The parasite observed at the earliest date was given a rank of 1, second a rank of 2, etc. If a host was re‐infected with a parasite, we used only the first date of infection with that species to calculate its rank. These observed ranks were compared with the predicted ranks generated from the nestedness analyses from either the combined 2009–2011 dataset or 2012 dataset with the degree of correlation between them measured by Spearman's Rho (*r*_s_). We compared observed and predicted ranks of infection for all parasites from the two datasets to test whether any patterns were generalizable enough to be consistent across years. Low p‐values (*p* < .05) from the Spearman's Rank analysis indicate a statistically significant concordance between predicted and observed rank orders, implying the ability to predict the order of within‐host community assembly from the results of a nestedness analysis of the whole host population.

For a finer resolution and more robust analysis of the temporal orders of infection, we used multi‐state Markov models (MSM) of longitudinal order of infection to test whether infection by one parasite species tended to occur after prior infection by another species. MSMs use a maximum likelihood approach to quantify the rates or probabilities of individuals transitioning between different observable states (Meira‐Machado, de Uña‐Álvarez, Cadarso‐Suárez, & Andersen, 2009), in our case, each state corresponds to host infections. This approach is more powerful that other statistical approaches, such as general linear models (Fenton, Knowles, Petchey, & Pedersen, 2014) due to its use of longitudinal data to parameterize the likelihoods of one infection following another, not simple associations between infections. This analysis also assumes individuals transition between states in continuous time and estimates each transition likelihood while taking into account all other possible likelihoods, as defined in the model, via a transition probability matrix (Jackson, 2011). This MSM approach has been used to study chronic disease progression in humans (Hoogenveen, van Baal, & Boshuizen, 2010; Huszti, Abrahamowicz, Alioum, & Quantin, 2011), but is starting to be used in ecological applications (Blackwell et al., 2013). We emphasize that no mechanisms are implied in this analysis, which simply quantifies whether the likelihood of a host transitioning to a state of being infected with one parasite species is more, less, or equally likely if they had been previously infected with another parasite species, compared to previously being uninfected. In other words, it provides a robust quantification of infection order (i.e., whether parasite B tends to infect before or after parasite A) among the parasite species tested. While this approach is more powerful than other forms of analysis, it requires very large datasets to parameterize all possible transitions between infection and coinfection states (Sofonea, Alizon, & Michalakis, 2015), so we restricted the models to the three most prevalent parasite species in the datasets and analyzed transitions in infection and coinfection status between all possible pairs of these three parasites. MSMs were carried out with the 2012 data only, as this dataset had better longitudinal records from individual hosts, as grids were sampled every two weeks compared with every four weeks, which is needed for the calculation of transition likelihoods.

**Figure 2:**
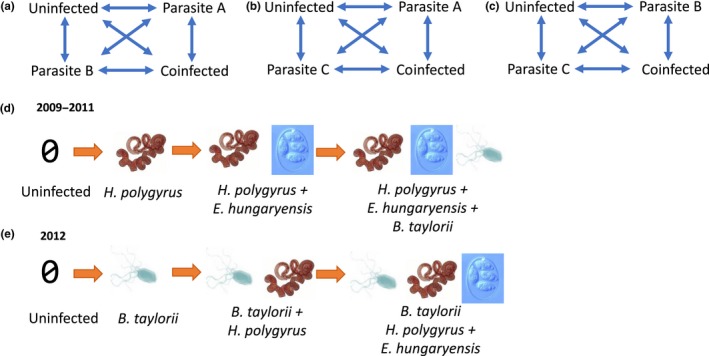
(a–c) Illustration of all possible pairwise infection transitions in the MSM analyses; (d) the predicted order of infection (community assembly) based on the nestedness analysis of the 2009–2011 dataset; and (e) the predicted order of infection based on the analysis of the 2012 dataset

We ran the MSMs using the *msm* R package (Jackson, 2011) to quantify the transition intensity, or likelihood, of hosts transitioning between infection states per unit time (days). This intensity is the “instantaneous risk” of the host moving from one infection state into another given infection state (Jackson, 2011, p. 1). Using all possible pairs of the three most common parasite species, hosts were assigned to one of four infection states at each capture: uninfected with either parasite, infected with parasite A, infected with parasite B, or coinfected with parasites A and B (Figure 2a‐c). To determine whether infection with one parasite is more likely to occur after prior infection with another, we compared the likelihood of host transitioning from an uninfected state to an infected state with a given parasite, compared to the transition from a singly infected state to the coinfected state. Transition intensities between infection states in each of the three pairs of parasites were compared to the predicted order of infection for these parasites from the nestedness analysis. All possible transitions were allowed to occur between consecutive time points, meaning a host could gain or lose one or both parasites in any one transition (Figure 2a‐c). Starting conditions for the model were estimated from the data (using the function “crudeinits.msm”) since we did not have prior assumptions about transition intensities. Transition intensities and 95% confidence intervals are presented. Sample size limitations did not allow for the addition of covariates in the MSM models. To assess the generality of parasite assembly rules in this community, we compared the observed order of infection to both to the predicted order of infection from the same year's (2012) population‐scale nestedness results as well as those from 2009 to 2011.

## 3 RESULTS

### 3.1 Population‐scale nestedness

The nestedness analysis of parasite community structure was first conducted on 1,352 individual wood mice sampled from 2009 to 2011 (2009, *n* = 441; 2010, *n* = 403; 2011, *n* = 508) and separately on the 322 mice from 2012. The most common parasites were the gut nematode *H. polygyrus*, multiple species of the gut apicomplexan coccidial protozoans in the genus *Eimeria* (*E. hungaryensis* and *E. apionodes*), and vector‐borne bacteria in the genus *Bartonella* (*B. taylorii* and *B. grahamii*). Also, present were other species of gut nematodes, cestodes, and less common *Bartonella* and *Eimeria* species (Table 1). The prevalence of each parasite differed across years; for example, infection prevalence of cestodes increased from 2009 to 2011 to 2012 (Table 1), whereas *H. polygyrus*, *Eimeria*, and *Bartonella* species were always highly prevalent. The majority of mice were infected with at least one parasite, 84% of individuals in the 2009 to 2011 dataset and 82% in the 2012 dataset.

The wood mouse parasite community in the 2009–2011 dataset was significantly more nested than expected when compared to a completely randomised community (Null model 1; SES = 77.248, *p* = 0.009), suggesting that the parasite community structure is indeed nonrandom (Figure 1a, Table S1). The parasite community for each individual year, 2009–2012, was also more nested than a completely randomly assembled community (Figure 1b, Figure S3, Table S1).

When we analyzed community structure while maintaining individual host species richness (row totals within the matrix kept constant), the observed community was also significantly more nested than the null (Null model 2; SES = 337.08, *p* = 0.009). In contrast, when the overall prevalence for each parasite was maintained (column totals within the matrix kept constant), the observed degree of nestedness was not significantly different from the null (Null model 3; SES = −0.072, *p* = 0.960). Results of analyzing each year separately showed the same patterns and significance (Table S1). Adults had richer parasite communities compared with young hosts, (Young mouse mean richness = 1.48 ± 0.05, median = 1, max = 7; Adult mouse mean richness = 1.98 ± 0.045 *SE*, median = 2, max = 7); however, both age classes contained nested communities and showed the same patterns of significance as the tests on the whole host population (Figure S4, Table S1). The parasites present in the young hosts did not appear to be a subset of those present in adults; young hosts could be infected with all parasites that infect adult hosts. These results suggest that while parasite communities within individual hosts are nonrandom, variation in parasite species prevalence is likely driving this pattern, not individual host‐level processes.

### 3.2 Individual‐scale community assembly

The above analysis suggests that wood mouse parasite communities are nested across the host population and that the degree of nestedness is related primarily to differences between parasites, rather than differences between hosts. As explained in the Methods section (see also Supporting Information; Figure S2), we hypothesized that individual‐scale parasite communities would assemble in accordance to their ranks in the nestedness matrices. To test this, we analyzed the individual‐level longitudinal data, first using rank order analyses of all parasite species used in the nestedness analyses, then using multi‐state Markov models of the three most prevalent species.

**Figure 3:**
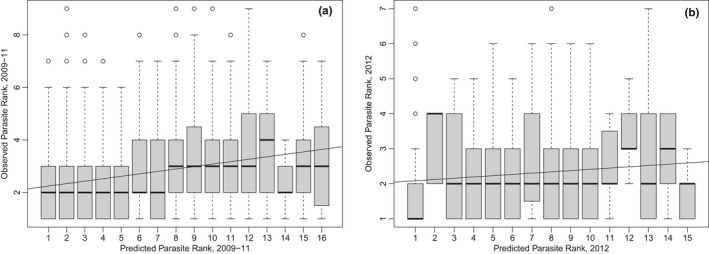
Concordance of predicted and observed parasite ranks from (a) 2009 to 2011 and (b) 2012. Predicted parasite ranks are along the *x*‐axis, observed ranks (order of infection within individual hosts) are along the *y*‐axis. Boxplots illustrate the distribution of observed ranks for the predicted rank of each parasite (median, interquartile range). The black line illustrates the linear relationship between predicted and observed ranks

The Spearman's Rank analysis, which tested the concordance between each parasite's predicted rank order of infection, from the nestedness analysis at the host population scale (Table 2), against the observed rank order of infection of each parasite at the individual scale, revealed a significant positive relationship between the predicted and observed rank orders of parasite infection (for all comparisons *p* < .01, except when testing the relationship between the predicted ranks from 2012 and observed data from 2009 to 2011; Table 3). Hence, there is evidence for some ability to predict individual‐level assembly order from patterns of nestedness at the population level. However, Spearman's r_s_ correlation values were relatively low, with between 2% and 12% of the variation in observed ranks being explained by predicted ranks (Figure 3, Table 3).

**Table 2:** Predicted ranks, derived from the results of the nestedness analyses, used in Spearman rank analyses to compare to observed parasite rank order of infection in each individual wood mouse host

Parasite	Predicted ranks	
	2009–2011	2012
Unknown *Aspicularis* sp.	14	11
*Bartonella birtlesii*	8	3
*Bartonella doshiae‐like*	11	7
*Bartonella grahamii*	5	6
*Bartonella taylorii*	3	1
*Bartonella* type BGA	13	8
unknown *Capillaria* sp.	12	12
unknown Cestodes	10	2
*Eimeria apionodes*	4	5
*Eimeria hungaryensis*	2	4
*Eimeria uptoni*	16	NA
Unknown *Eimeria* sp. 1	6	15
Unknown *Eimeria* sp. 2	15	14
*Heligmosimoides polygyrus*	1	13
*Syphacia stroma*	9	10
*Trypanosoma grosi*	7	9

**Table 3:** Results of Spearman rank analyses. Results presented are those of the observed ranks (order in which a host was infected with each parasite) compared to the predicted ranks from either the same dataset (e.g., 2012 predicted ranks and 2012 observed ranks) or different dataset (e.g., 2012 predicted ranks and 2009–2011 observed ranks). Both comparisons were done to test the generality of the predictions generated from each dataset

	Predicted ranks, same dataset		Predicted ranks, different dataset	
	*r*_S_	*p*‐value	*r*_S_	*p*‐value
2009–2011	0.177	<0.0001	0.023	0.263
2012	0.110	0.011	0.120	0.006

The MSMs used the three most prevalent parasite species from all years of data collection: gastrointestinal parasites *H. polygyrus* (33% infection 2009–2011, 22.4% 2012) and *E. hungaryensis* (28.4% infected 2009–2011, 18% 2012), which have been found to interact within coinfected mice (Knowles et al., 2013), and the flea‐transmitted, blood‐borne bacterium *B. taylorii* (23.3% infected, 31.7% 2012; Withenshaw et al., 2016). Hence, the predictions for individual‐level community assembly based on the population‐level nestedness analysis using 2009–2011 data were: Uninfected → *E. hungaryensis* → *E. hungaryensis* + *H. polygyrus* → *E. hungaryensis* + *H. polygyrus* + *B. taylorii* (Figure 2d); predictions from the 2012 data were: Uninfected → *B. taylorii* → *B. taylorii + H. polygyrus* → *B. taylorii + H. polygyrus* + *E. hungaryensis* (Figure 2e). The pairwise associations tested with the MSMs were: *H. polygyrus‐E. hungaryensis*,* H. polygyrus‐B. taylorii*, and *E. hungaryensis‐B. taylorii*. Then, using the 2012 dataset, we tested whether the outcome of the analyses of parasite community assembly at the individual host scale was consistent with the predictions from the nestedness analyses at the host population scale.

Contrary to our predictions, none of our MSM analyses revealed cases where an individual was more likely to become infected with a parasite after previously being infected with a different parasite species, compared to becoming infected from an uninfected state (Table 4). For example, in the 2012 dataset, the parasite with the first nestedness rank and highest prevalence was *B. taylorii* which would therefore be expected to be the parasite most likely to infect first. However, uninfected hosts were more likely to become infected with *E. hungaryensis* (0.036, 95% CI: 0.002, 0.892) from an uninfected state compared with *B. taylorii* (0.017, CI: 0.010, 0.030), and equally likely to become infected with *H. polygyrus* (0.017, CI: 0.009, 0.033) compared with *B. taylorii* (0.017, CI: 0.009, 0.032). To compare to the 2009–2011 predictions, *H. polygyrus* was the most prevalent parasite and would therefore be expected to be the first to infect. However, uninfected hosts were more likely to become infected with *E. hungaryensis* first (0.033, CI: 0.002) compared with *H. polygyrus* (0.007, CI: 0.0001, 0.317), and, as stated above, hosts were equally likely to become infected with either *H. polygyrus* or *B. taylorii* from an uninfected state. Hence, while parasite abundance seemed to be the driving mechanism structuring parasite communities at the host population scale, the inconsistency of infection order from these individual‐scale results suggests there is not a deterministic order of infection among these three parasites.

**Table 4:** Transition likelihoods with confidence intervals for all pairwise multi‐state Markov (MSM) infection models. Hosts were able to transition between any two states per unit time (day). If a transition likelihood is “0”, this is due to there being no records of a host transitioning between those two states in the dataset

		To			
**From**	***E. hungaryensis—H. polygyrus***				
		**Uninf**	***E. hungarensis* only**	***H. polygyrus* only**	**Coinfection**
	**Uninf**	0.004 (0.345,0.006)	0.033 (0.002, 0.071)	0.007 (0.0001, 0.032)	0.004 (0.001, 0.011)
	***E. hung* only**	0.023 (0.009, 5.847)	0.028 (5.796,0.014)	0.050 (0.001, 2.666)	0
	***H. poly* only**	0.040 (0.020, 0.076)	0.000006 (0.011e−82, 2.949e+71)	0.043 (0.083, 0.023)	0.004 (0.0002, 0.066)
	**Coinfection**	0.017 (0.001, 0.021)	0	0.041 (0.011, 0.151)	0.057 (0.129,0.025)
	***B. taylorii—E. hungaryensis***				
		**Uninf**	***B. taylorii* only**	***E. hungarensis* only**	**Coinfection**
	**Uninf**	0.054 (0.468, 0.006)	0.017 (0.010, 0.030)	0.036 (0.002, 0.892)	0.0001 ( 5.044e−10, 1.392e+01)
	***B. taylorii* only**	0.0397 (0.019, 0.083)	0.0681 (0.145,0.032)	0.001 (9.770e−12, 2.359e+04)	0.028 (0.006, 0.128)
	***E. hung* only**	0.312 (0.013, 7.635)	0.003 (2.528e−09, 2.780e+03)	0.315 (7.488, 0.013)	0.0006 (7.906e−10, 3.858e+02)
	**Coinfection**	0.005 (1.835e−08, 1.178e+03)	0.106 (0.012, 0.572)	0	0.111+e13 (0.469,0.026)
	***B. taylorii—H. polygyrus***				
		**Uninf**	***B. taylorii* only**	***H. polygyrus* only**	**Coinfection**
	**Uninf**	0.038 (0.059, 0.024)	0.017 (0.009, 0.033)	0.017 (0.009, 0.033)	0.003 (4.525e−04, 0.026)
	***B. taylorii* only**	0.030 (0.016, 0.056)	0.035 (0.059, 0.021)	0.0004 ( 1.538e−08, 12.887)	0.005 (8.085e−04, 0.033)
	***H. poly* only**	0.037 (0.017, 0.082)	0.001 (9.684e−08,17.118)	0.040 (0.075,0.021)	0.001 (1.642e−06, 1.276)
	**Coinfection**	0.017 (4.501e−04, 0.655)	0.013 (7.371e−04, 0.229)	0.023 (0.003, 0.203)	0.053 (0.013, 0.022)

## 4 DISCUSSION

By combining analyses across scales, from host population to individual, we show (a) that there is clear nonrandom structure to the parasite communities of wild wood mice, (b) this nonrandomness is not related to systematic differences between hosts, and (c) this observed structure does not translate to predicting within‐host–parasite community assembly over time. Overall, our results do not provide evidence for patterns at one scale directly predicting processes at another in this system, suggesting the observed patterns of community structure may be arising from neutral or stochastic processes. We suggest targeted experiments are needed to fully elucidate the intrinsic and extrinsic mechanisms behind such observed patterns of parasite community structure (Boughton, Joop, & Armitage, 2011; Pedersen & Fenton, 2015).

Our population‐scale analyses showed that parasite communities are highly nested across hosts, such that species‐poor parasite communities (i.e., hosts with relatively few coinfecting species) tended to be subsets of species‐rich parasite communities (i.e., hosts harboring many coinfecting species). Hence, there tended to be highly prevalent “core” parasite species found in most communities, and less prevalent “satellite” species found in fewer communities (Bush & Holmes, 1986; Holmes & Price, 1986; Stock & Holmes, 1987). Furthermore, we showed that parasite species identity, rather than factors relating to host identity, appeared to be the key driver of the observed degree of nestedness. Community ecology theory predicts four mechanisms generally drive community nestedness: selective colonization among species, selective extinction, habitat nestedness, or neutral, stochastic sampling (Atmar & Patterson, 1993; Azeria, Carlson, Part, & Wiklund, 2006; Ulrich et al., 2009). Given we found no signal of host‐related factors driving the observed nestedness, we focussed on processes relating to the colonization process (i.e., the acquisition of infections) in driving these patterns. In particular, we tested the hypothesis from free‐living community ecology (Atmar & Patterson, 1993; Diamond, 1975; Ulrich et al., 2009) that observed nestedness arises from a fixed, predictable order of colonization (infection order). Epidemiological theory, supported by our simulations (Supporting Information, Figure S2), predicts there should be an inverse relationship between population‐level prevalence and order of infection (parasites with higher population prevalence have shorter times to first infection in an individual host; Anderson & May, 1991), thereby providing an explicit link between patterns of parasite community structure at the host population scale with the process of parasite infection order at the individual host scale. However, we found very little support for a relationship between the order of infection among individual mice and the predictions arising from the nestedness analysis. We also did not observe young hosts to have a less rich subset of the parasite communities observed in older, adult mice. Together, these results suggest that although parasite community composition at the host population scale is driven by parasite species‐specific variation, parasite community assembly within individual hosts is not predictable from population‐scale analyses.

Given the lack of predictability in infection order, our results suggest that neutral or stochastic processes may be generating the levels of community nestedness observed (Higgins, Willig, & Strauss, 2006; Ulrich et al., 2009; Ulrich & Gotelli, 2013). Some aspects of this system, such as differences in parasite infection prevalence among years, suggest that there is natural variation in the force of parasite infection, which will likely impact both host exposure and the likelihood of successful infection. We acknowledge that while we have parasites that span a range of taxonomic groups and transmission modes, we do not have data on parasite variables outside of the hosts, such as the infection prevalence in vectors or abundance of infectious stages in the environment. An important next step in assessing the extrinsic mechanisms that impact parasite community structure would be to integrate these system‐specific details integral to each parasite's life cycle. In addition, we focused on species gains in our analysis of parasite community assembly, but species losses, through processes such as host clearance of infection, are also an important factor driving parasite community patterns. It is likely that parasite communities experience succession‐like dynamics, with early and late colonizers, which would compete for habitat and resources, with some species ultimately being lost in this process (Rynkiewicz et al., 2015). However, from our longitudinal sampling we rarely observe the loss of any of these parasites from an individual. Of course, there may be losses followed by reinfection occurring, but these are difficult to distinguish with the resolution of sampling methods used here. So, while we focused on colonizations (gains of infection) rather than losses, we acknowledge that integrating both would be needed to differentiate these processes in future analyses.

As stated above, our results found no evidence for host‐related factors playing a significant role in shaping parasite community structure. One explanation for this is that host‐level processes that were not measured in our study may influence the outcome of parasite community assembly. Variation in an individual host's immune response to infection, or cross‐reactivity between parasite‐specific antibodies, may determine the outcome of a parasite infection in an individual host (Cobey & Lipsitch, 2013; Graham, Cattadori, Lloyd‐Smith, Ferrari, & Bjornstad, 2007). The dynamic nature of the host immune response may lead to fluctuations of the within‐host immune environment, such as switching between being dominated by inflammatory or anti‐inflammatory components, in shorter periods of time than the sampling regime used in the longitudinal dataset. For example, after infection with *H. polygyrus* laboratory, mice show a shift toward an anti‐inflammatory immune profile in a matter of days (Monroy & Enriquez, 1992). Finer‐scale monitoring of the host's response to parasite infection or experimental manipulation of the immune response could better describe these interactions to further investigate within‐host processes as mechanisms impacting community structure.

Our analyses quantified infections in terms of their presence or absence; it may be, however, that there are more subtle, quantitative effects driven by variation in infection burdens. Wild host populations show significant variation in parasite burdens (Shaw & Dobson, 1995), and extrinsic factors, such as resource availability (Budischak et al., 2015; Pedersen & Greives, 2008; Ramiro, Pollitt, Mideo, & Reece, 2016), as well as intrinsic factors, such as immune phenotype (Cobey & Lipsitch, 2013; Reese et al., 2014), can impact host–parasite interactions and coinfection susceptibility. This could mean that analyses that include only parasite presence (i.e., whether a host is infected or uninfected) may not fully describe the interaction between host and parasite. Furthermore, theory suggests that the magnitude, and even direction (net positive or negative) of within‐host–parasite interactions can vary depending on the burden of infection (Fenton, 2013). As such it may be that our analyses using infection status, and not burdens, are too coarse to detect any signal of prior, burden‐dependent infection by one parasite species on subsequent infection by another. However, most data collected on wild parasite infections are in the form of presence/absence, therefore, there are practical reasons to test the ability, or inability, of these sorts of data to inform community processes at multiple scales.

Overall, we found little evidence for deterministic assembly order at the individual‐scale driving the observed nonrandom structure seen in the wild wood mouse parasite communities. While there is a growing appreciation that ecological tools and concepts developed for free‐living communities can be applied to understanding the hierarchical nature of host–parasite communities, there are still many challenges to successfully integrating ecological information among scales (Handel & Rohani, 2015; Johnson et al., 2015; Sofonea et al., 2015). Practically, most data collected from parasite–host systems are cross‐sectional and are often used to make predictions of disease dynamics at scales beyond the original individual, population, or community scale at which it was originally collected. Our results show this can lead to false or spurious conclusions concerning individual‐level parasite community assembly. The diversity of combinations of extrinsic and intrinsic processes means that trying to infer what mechanisms drive the interactions at one scale, such as the impacts of competing parasites in coinfected individuals, from patterns occurring at another scale, such as the force of infection driving parasite prevalence, is difficult and more research is needed to understand the connections between these ecological processes across multiple scales. We suggest the best approach to deal with these complexities is to integrate data from the same system at multiple scales with experiments to directly elucidate the directionality of processes at one scale and their consequences at another. Further utilization of host–parasite systems as models for community assembly will be a critical tool in this pursuit.

## CONFLICT OF INTEREST

None declared.

## AUTHORS’ CONTRIBUTIONS

ECR and ABP conceived of the idea for the study and developed the methodology along with AF. ECR conducted the data analyses and production of figures. AF developed the simulation model. ECR led the writing of the manuscript but all authors contributed significantly to its writing. All gave final approval for publication.

## Data Availability

All data associated with this study have been deposited in the Dryad Digital Repository (https://doi.org/10.5061/dryad.n02v6wwsd).
