# Supplementary material for: Linking community assembly and structure across scales in a wild mouse parasite community
Source: Ecol Evol. 2019 Dec 9;9(24):13752–63. doi: 10.1002/ece3.5785 (PMC6953566; doi:10.1002/ece3.5785)
Supplement: Supplementary file 1 [file ECE3-9-13752-s001.pdf]

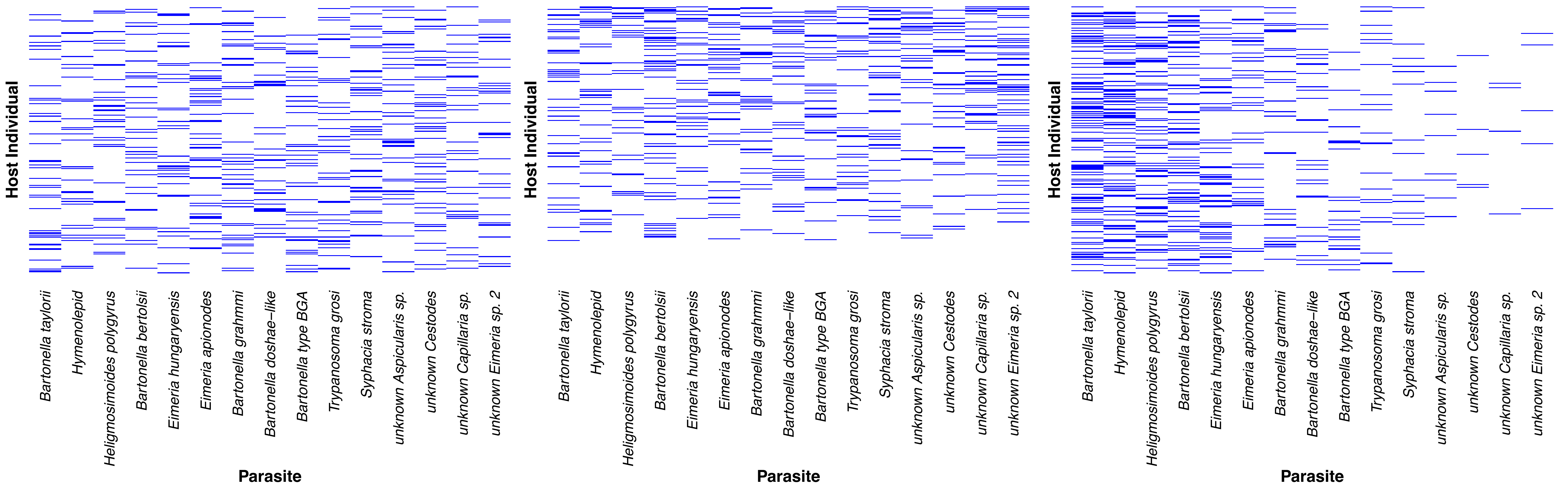

a) **Null Model 1**: Random b) **Null Model 2**: Host richness constant c) **Null Model 3**: Parasite abundance constant
