# Supplementary figures and images for: Linking community assembly and structure across scales in a wild mouse parasite community
Source: Ecol Evol. 2019 Dec 9;9(24):13752–63. doi: 10.1002/ece3.5785 (PMC6953566; doi:10.1002/ece3.5785)

Mean time to first infection of parasite i

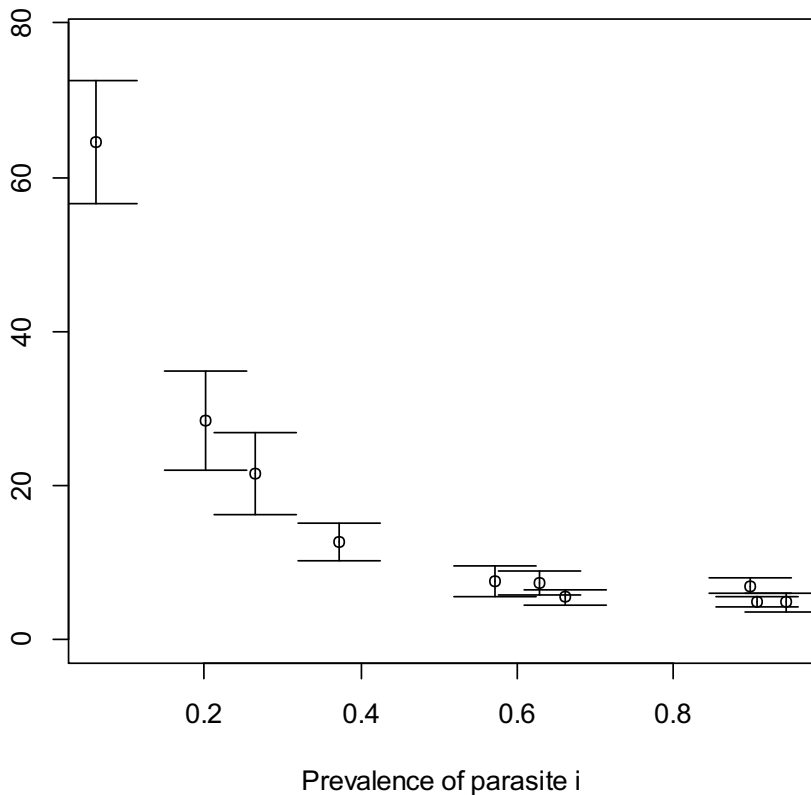

Supplement: Supplementary file 2 [file ECE3-9-13752-s002.pdf]

a) 2009

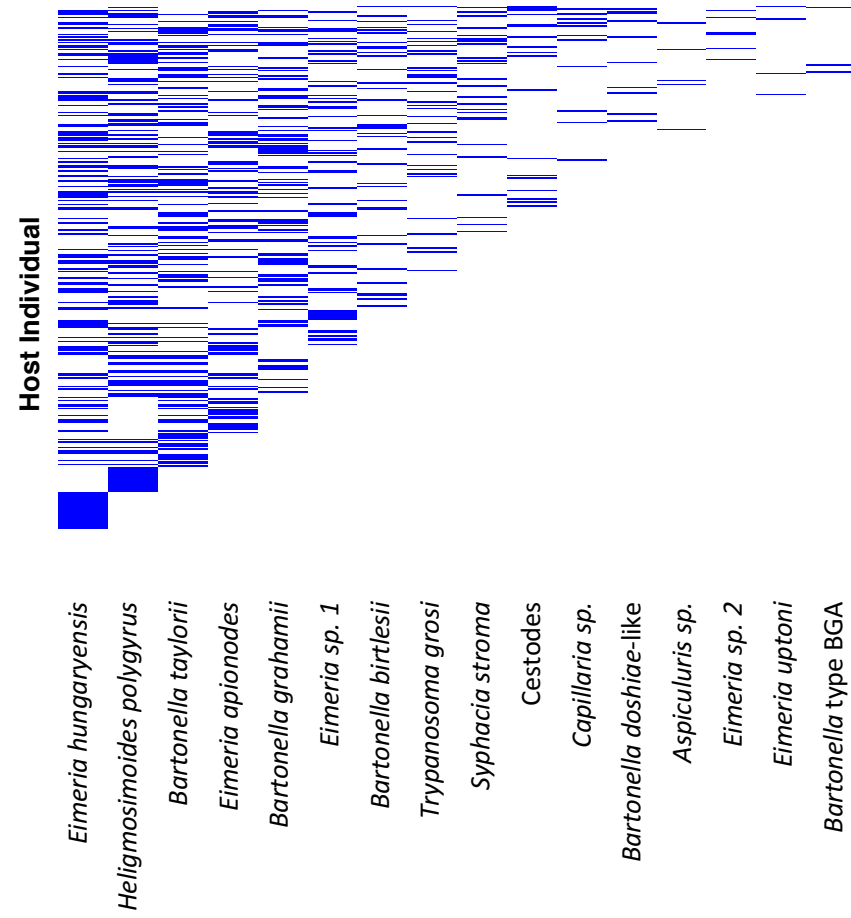

b) 2010

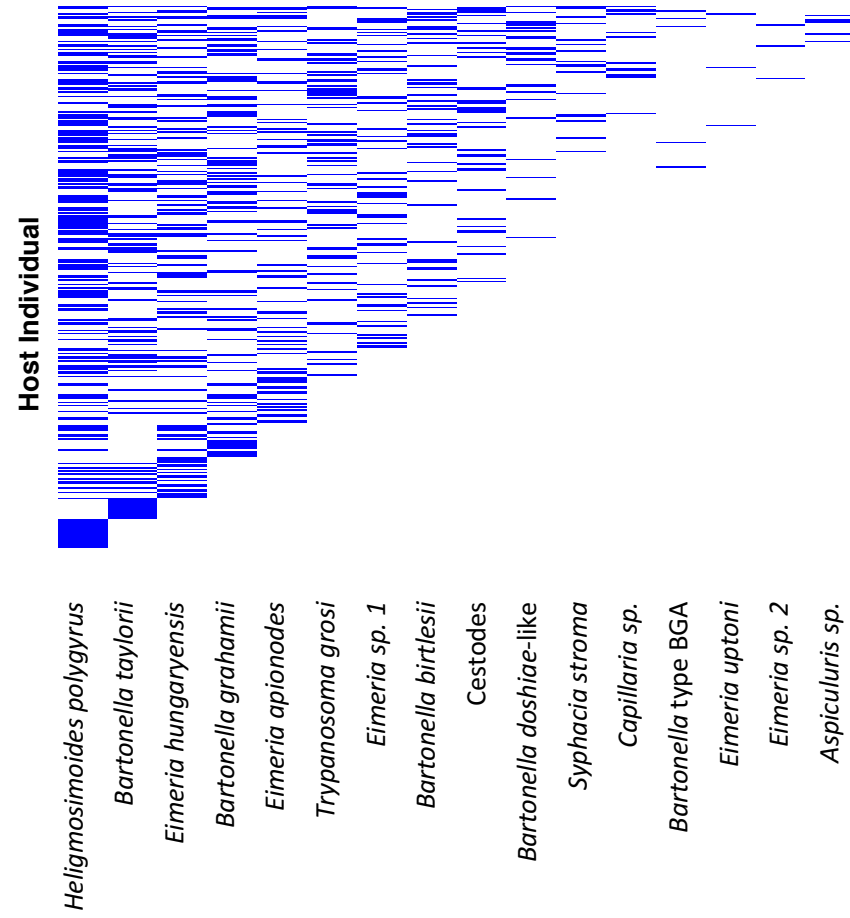

c) 2011

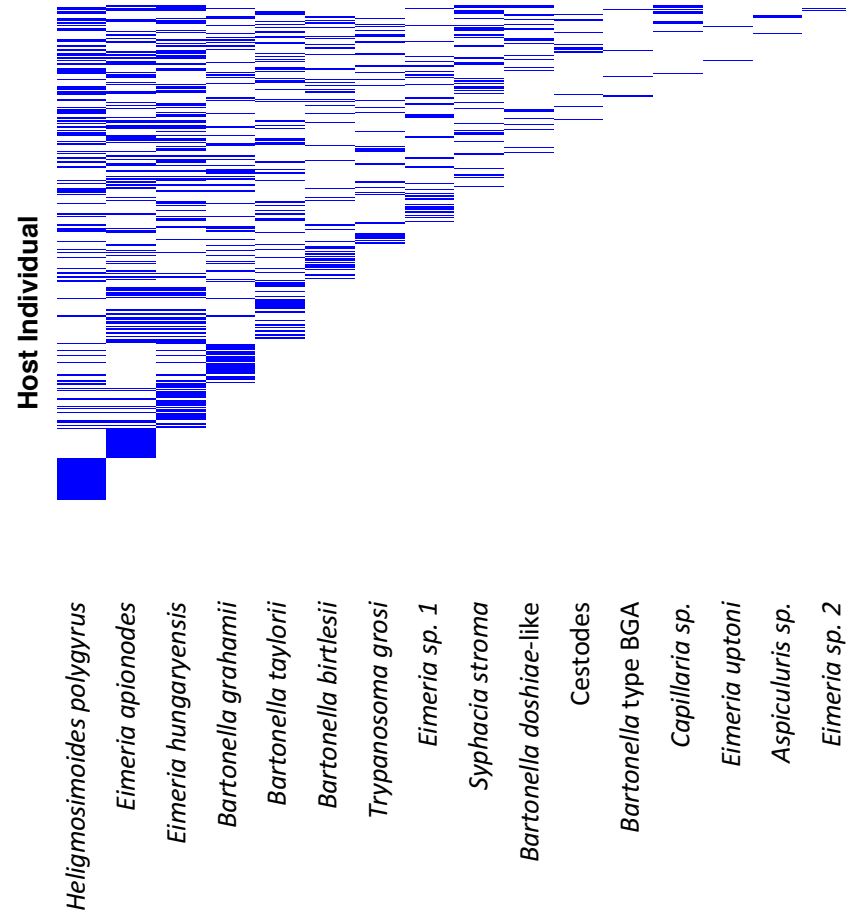

Supplement: Supplementary file 3 [file ECE3-9-13752-s003.pdf]

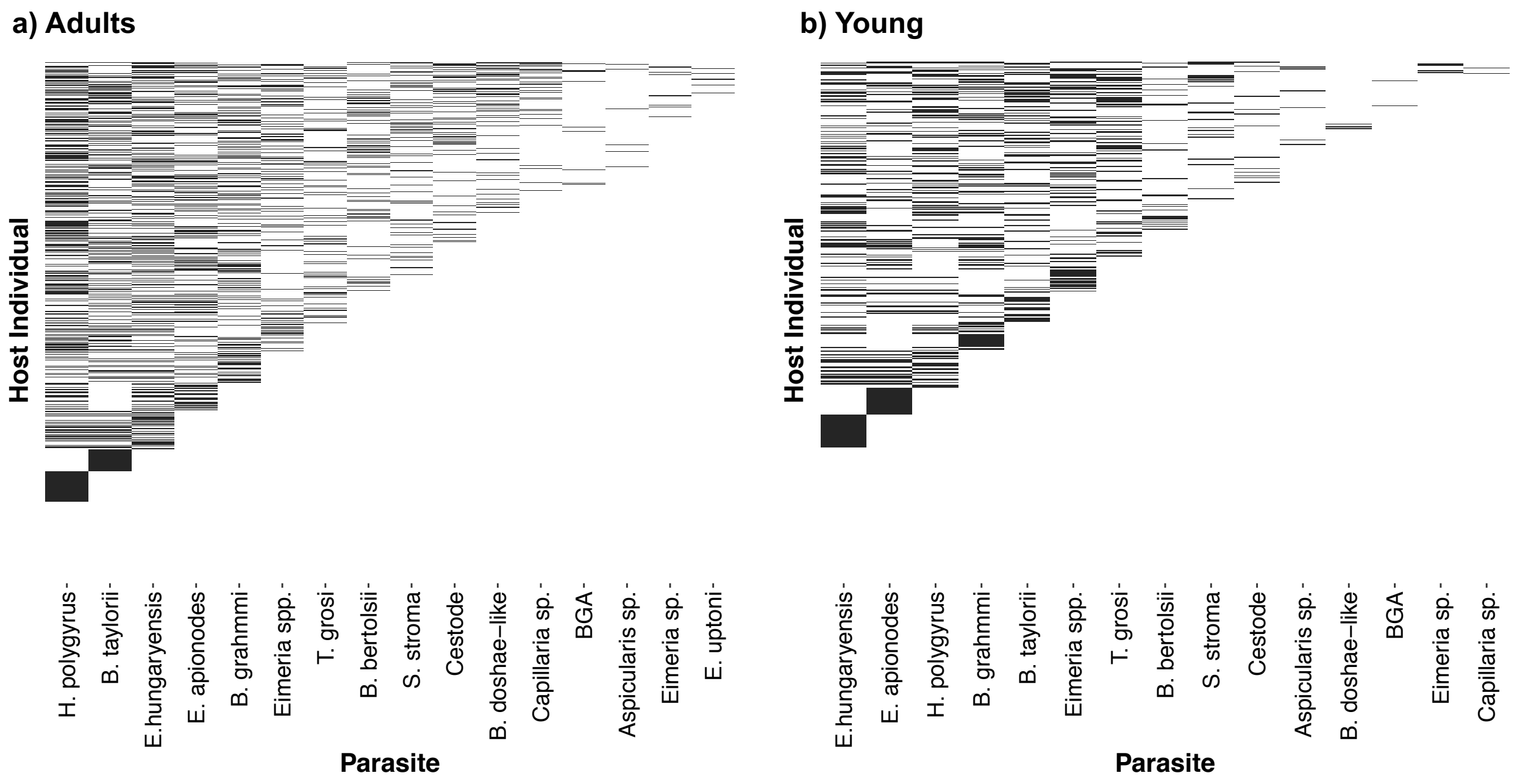

Supplement: Supplementary file 4 [file ECE3-9-13752-s004.pdf]
