# Supplementary material for: Linking community assembly and structure across scales in a wild mouse parasite community
Source: Ecol Evol. 2019 Dec 9;9(24):13752–63. doi: 10.1002/ece3.5785 (PMC6953566; doi:10.1002/ece3.5785)
Supplement: Supplementary file 6 [file ECE3-9-13752-s006.docx]

**Supporting Information**

*Simulation of linkage between parasite prevalence and order of infection*

Parasite characteristics measured at the host population scale, such as prevalence or abundance, are expected to be related to traits that impact how individual hosts become infected. These connections have been previously described, modelled, and tested in the disease ecology literature, showing that parasites with higher basic reproduction number (R_0_) will be more common (have higher prevalence) and infect a host faster (lower mean age at first infection) than parasites with lower R_0_ (Grenfell & Anderson 1985; Dobson 1990; Anderson & May 1991). Here we describe a stochastic simulation model to illustrate the relationship between population-level prevalence and individual-level infection order.

We assume the mean rate at which an individual host gets infected by parasite species *i* is:

$\frac{dp_{i}}{dt}= \bar{\beta}_{i}P_{i}H$ Eq 1

where $p_{i}$ is the number of new infections of parasite species *i* experienced by an average host individual. This depends on the parasite prevalence ($P_{i}$), the total number of hosts in the wider population (*H*, such that the total number of hosts infected with parasite *i* is $P_{i}$*H*), multiplied by a *per capita* contact-and-transmission parameter $\bar{\beta}_{i}$. The parameter combination ($\bar{\beta}_{i}P_{i}H$) therefore is the force of infection of parasite species *i* on the host ($\lambda_{i}$), representing the mean number of successful infections experienced by each host per unit time. For simplicity, we keep the *per capita* transmission parameter ($\bar{\beta}_{i}$) constant across all hosts and parasite species *i*, which allows us to focus on the impact of varying parasite prevalence on the rate at which hosts become infected. We rephrase this deterministic skeleton as a stochastic model by assuming the probability with which a given host becomes infected with a parasite *i* per unit time can be modelled as a Poisson process, using the parasite-specific force of infection ($\lambda_{i}$) as the rate parameter. We then simulated the accumulation of 10 species of parasite, which varied in their forces of infection based on their population-level prevalences, on 20 host individuals, recording the time to first infection of each host by each parasite species.

The model shows a negative relationship between population-level prevalence and mean time to first infection (Fig. S2). As such we would expect that a population-scale characteristic, prevalence, should predict the order of within-host parasite community assembly, such that ‘core’ parasite species which are present in most communities should infect sooner than rarer, ‘satellite’ species.

**References**

Anderson, R. & May, R.M. (1991) *Infectious Disease of Humans: Dynamics and Control*. Oxford University Press, Oxford.

Dobson, A.P. (1990) Models for multi-species parasite-host communities. *The Structure of Parasite Communities* (eds G. Esch, C. R. Kennedy & J. Aho), pp. 261-288.

Grenfell, B.T. & Anderson, R.M. (1985) The estimation of age-related rates of infection from case notigications and serological data. *Journal of Hygiene,* **95,** 419-436.

Table S1. Results of nestedness analyses of all datasets against the three null models: 1) fully random, 2) row totals (host parasite species richness) kept constant, 3) column totals (parasite abundance) kept constant.

|  | **Models:** | **Completely random** | | | | **Row totals constant** | | | | **Column totals constant** | | | |
| --- | --- | --- | --- | --- | --- | --- | --- | --- | --- | --- | --- | --- | --- |
| **Year** | **Matrix Temperature** | **SES** | **mean** | **p-value** | **SES** | | **mean** | **p-value** | **SES** | | **mean** | **p-value** |  |
| **2009-11** | 18.448 | 77.248 | 9.812 | 0.0099 | 337.08 | | 9.812 | 0.0099 | -0.072 | | 18.466 | 0.96 |  |
| **2009** | 19.579 | 35.6609 | 10.72 | 0.0099 | 111.929 | | 10.572 | 0.0099 | -0.951 | | 19.99 | 0.327 |  |
| **2010** | 23.059 | 45.649 | 12.54 | 0.0099 | 118.386 | | 12.45 | 0.0099 | -0.768 | | 23.529 | 0.426 |  |
| **2011** | 14.596 | 42.992 | 7.55 | 0.0099 | 111.936 | | 7.7 | 0.0099 | -0.246 | | 14.66 | 0.703 |  |
| **2012** | 18.085 | 25.135 | 11 | 0.0099 | 64.278 | | 10.955 | 0.0099 | 0.187 | | 17.096 | 0.7822 |  |
|  |  |  |  |  |  | |  |  |  | |  |  |  |
| **Adults (2009-11)** | 8.465 | 64.468 | 11.504 | 0.0099 | 263.503 | | 11.499 | 0.0099 | 0.19601 | | 22.201 | 0.8812 |  |
| **Young (2009-11)** | 4.476 | 50.132 | 7.277 | 0.0099 | 104.080 | | 7.291 | 0.0099 | 0.0279 | | 14.328 | 0.9208 |  |

Fig. S1. Nested matrix examples for each of the 3 null models used in the NODF analysis: a) random model (number of parasites total is the same, but they are assigned to parasites species and host individuals at random), b) host richness constant (host richness is identical to what is observed, parasite identity is randomized), c) parasite abundance constant (parasite species abundance constant, which hosts are infected is randomized). Each figure represents one of the 100 null communities generated from the, in this case, 2012 dataset for comparison to the observed nestedness of the wood mouse population.

Fig. S2. Results of the simulation exploring relationship between host population-scale parasite prevalence and time to first infection. Parasites with higher prevalence have shorter times to first infection, such that they are expected to infect an individual host faster than less-prevalent parasites.

Fig. S3. Nestedness plots for the parasite communities from the years 2009, 2010, and 2011 individually.

Fig. S4. Nestedness plots for a) adults and b) young hosts. Hosts in both age classes had similar levels of nestedness and did not show patterns of young hosts containing subsets of the parasites infecting older, adult hosts.
